# Massive adrenocortical carcinoma presenting as peripheral edema: a case report

**DOI:** 10.1186/s13256-022-03397-5

**Published:** 2022-06-20

**Authors:** David A. Goodkin

**Affiliations:** Washington, USA

**Keywords:** Adrenocortical carcinoma, Mitotane, Ki67 index, Peripheral edema

## Abstract

**Background:**

Adrenocortical carcinoma is a rare, but potentially lethal, malignancy that is usually detected as an incidental finding on abdominal imaging studies or owing to hormonal complications. This report recounts an unusual presentation with leg edema due to compression of the inferior vena cava. The dearth of proven effective treatment is also addressed.

**Case presentation:**

A 65-year-old White male physician presented with severe, bilateral pitting edema that extended from the toes to the thighs. It progressed over several months. He also experienced paroxysmal dyspnea. Evaluation of cardiac, hepatic, and renal function failed to determine a cause. Computed tomography revealed a tumor above the right kidney, with compression of the intrahepatic inferior vena cava and upstream distension. Serum cortisol and dehydroepiandrosterone sulfate concentrations were elevated, 24-hour urinary cortisol level was elevated, and serum adrenocorticotropic hormone and testosterone concentrations were suppressed. A 27-cm tumor, the right lobe of the liver, the right kidney, and 26 lymph nodes were resected. Histological study confirmed the diagnosis of adrenocortical carcinoma. Ki67 proliferation index was 26.7% (worse prognosis associated with index > 10%). Lymph nodes were negative for malignancy, but a separate 2.7-cm tumor was found near the renal hilum. Adjuvant mitotane chemotherapy was prescribed. Serum testosterone concentration returned to normal. High-dose hydrocortisone administration was needed because of adrenal suppression and CYP 3A4 induction by mitotane.

**Conclusion:**

Imaging of the abdomen and pelvis should be conducted in cases of unexplained leg edema. In this case, a large adrenal cancer compressed the vena cava. Iron deficiency followed resection of the large tumor. Advanced stages of adrenocortical carcinoma are associated with poor prognosis. Mitotane chemotherapy is a standard but unproven adjuvant treatment that is associated with many complications, and its induction of hepatic CYP 3A4 enzymes necessitates adjustment of other medications.

## Introduction

Peripheral edema is often caused by diminished cardiac output, sodium and water retention due to kidney or liver failure, decreased serum albumin concentration (for example, due to nephrotic syndrome), or medications such as vasodilators, nonsteroidal anti-inflammatory drugs, or steroid hormones [1, 2]. Edema due to cardiac failure is typically most extensive in the legs, whereas hypoproteinemic edema tends to be more generalized and may manifest notably in the face [1]. After these common etiologies have been excluded, clinicians should consider obstruction of venous blood return to the heart.

This case report describes an unusual case of peripheral edema caused by constriction of the inferior vena cava by a solid tumor high in the abdomen. The differential diagnosis of less common, obstructive causes of chronic bilateral leg edema in the literature typically alludes to pelvic malignancy or lymphoma, but not abdominal solid tumors [3]. Also of note in the current case is the importance of verified, full communication between radiologists, emergency department physicians, and patients. A small (1.2 cm) adrenal tumor that could easily have been removed for cure was detected as an incidental finding during evaluation of renal colic 9 years prior to the current diagnosis of a 27-cm adrenal cancer with a satellite lesion, but the full radiologic report that followed the individual’s trip to the emergency department did not prompt further evaluation.

## Case presentation

A 65-year-old White male physician experienced accumulation of bilateral leg edema over several months, accompanied by episodes of paroxysmal dyspnea. The edema was pitting. Initially it was evident only over the feet and ankles, but the swelling then spread upward above the knees. There was no redness, tenderness, or palpable mass. The patient had been in generally good health, suffering only hypercholesterolemia well controlled with atorvastatin, mild exercise-induced asthma prevented by montelukast, and a prior episode of nephrolithiasis. He had no chest pain, orthopnea, hemoptysis, or other signs or symptoms of heart, liver, or kidney disease. There was no history of unusual environmental exposures or family history of adrenal malignancy. The patient did not smoke and rarely consumed alcohol. Vital signs were within normal ranges, and physical examination was unremarkable with the exception of the edema. Evaluations of the cardiorespiratory system (including electrocardiogram, chest radiography, stress echocardiography, spirometry, and carbon monoxide diffusing capacity), hepatic function, and renal function were normal. Serum albumin concentration and routine blood and urine studies were normal. The edema improved with compression stockings and diuretics.

**Fig. 1:**
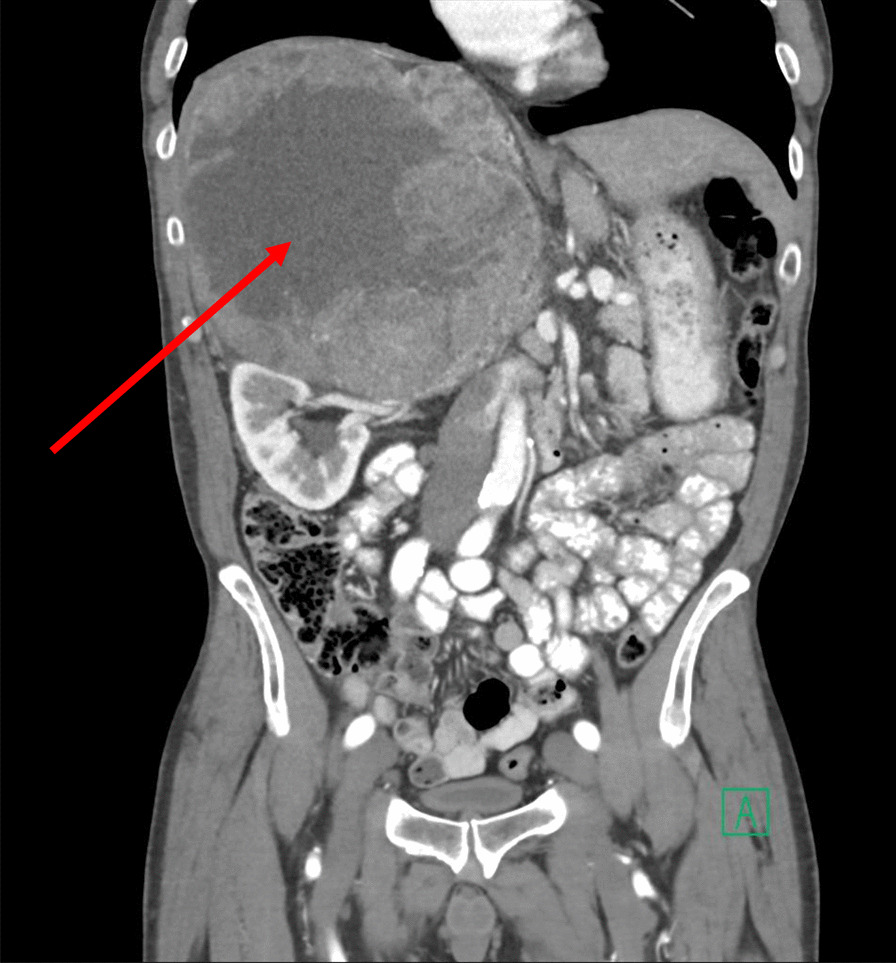
Computed tomography of the abdomen and pelvis. Arrow: adrenocortical carcinoma

Computed tomography revealed a large tumor above the right kidney (Fig. 1, arrow) with compression of the intrahepatic inferior vena cava and upstream distension. Serum cortisol (18.0 µg/dL, normal < 11.3 µg/dL), 24-hour urinary cortisol (73 µg, normal 3.5–45 µg), and serum dehydroepiandrosterone sulfate (734 µg/dL, normal 35–212 µg/dL) levels were elevated, consistent with a hormonally active adrenal neoplasm. Serum adrenocorticotropic hormone (< 5.0 pg/mL, normal 7.2–63 pg/mL) and free testosterone (28 pg/mL, normal 30–140 pg/mL) concentrations were suppressed. Blood and urine studies of metanephrines, estradiol, renin, and aldosterone were normal. Positron emission tomography and chest computed tomography did not detect metastatic disease.

**Fig. 2:**
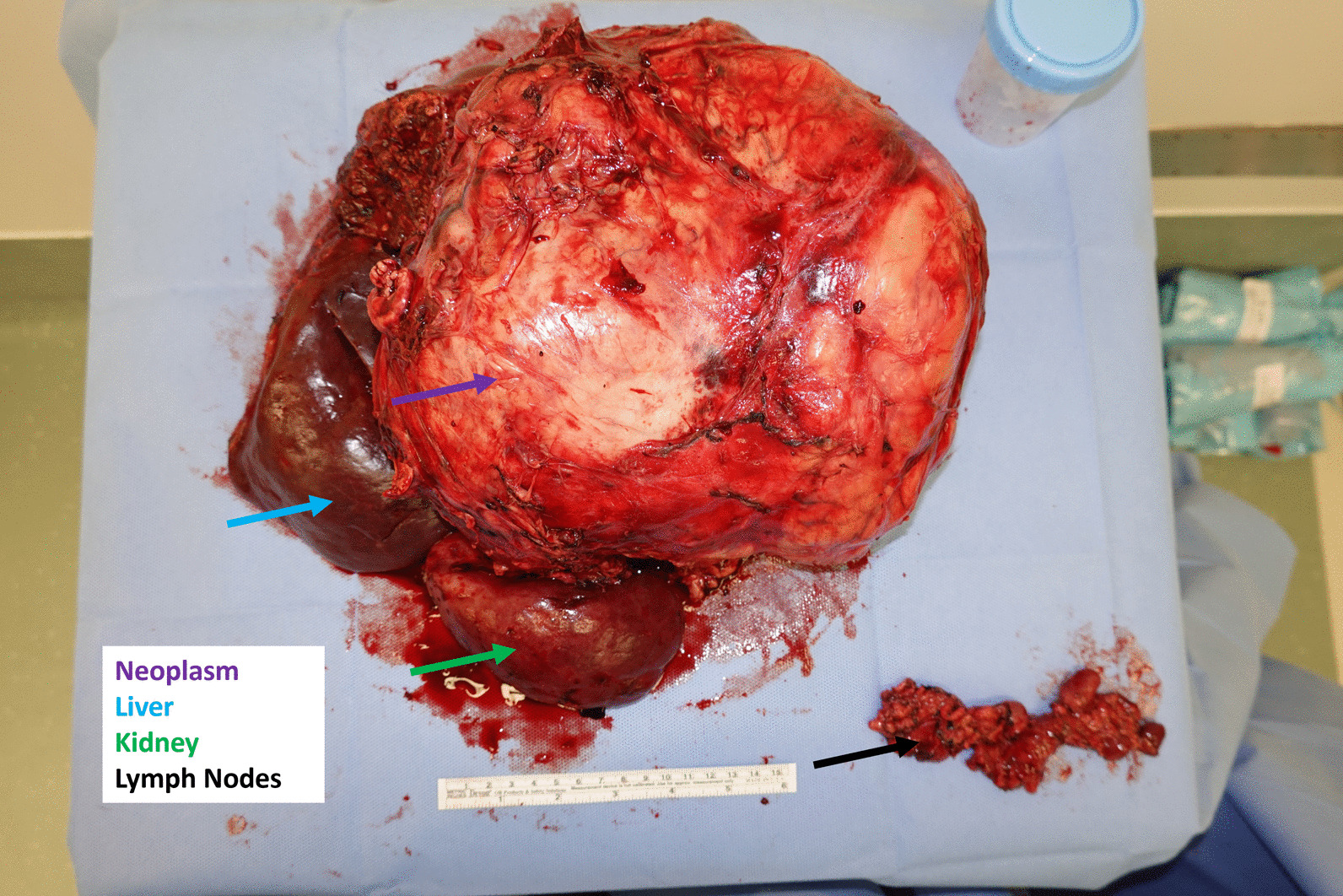
Surgical specimens: adrenal tumor, right lobe of the liver, right kidney, and lymph nodes. The arrows are color coded to match the labels (ie, purple=neoplasm; blue = liver; green = kidney; black = lymph nodes)

A 27-cm tumor, the right lobe of the liver, the right kidney, the gallbladder, and 26 lymph nodes were resected (Fig. 2). A separate 2.7-cm tumor was found near the renal hilum. The cancer was stage 3, based on size and local extension. The postoperative course was complicated by *Clostridioides difficile* enterocolitis, multidrug-resistant urinary tract infection, and intraabdominal chyle leak (following lymph node resection) that prompted parenteral nutrition for a week.

**Fig. 3:**
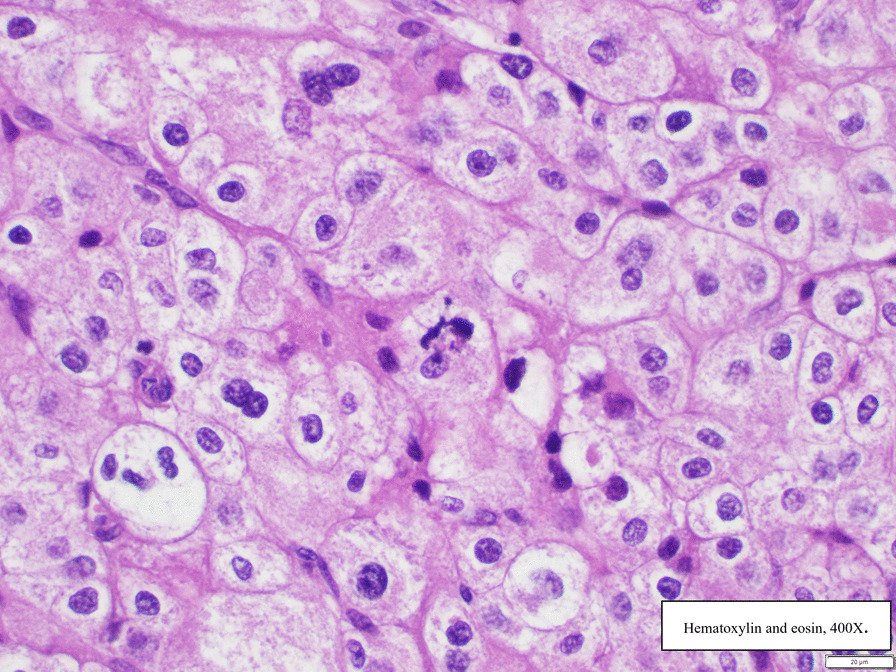
Tumor histology

**Fig. 4:**
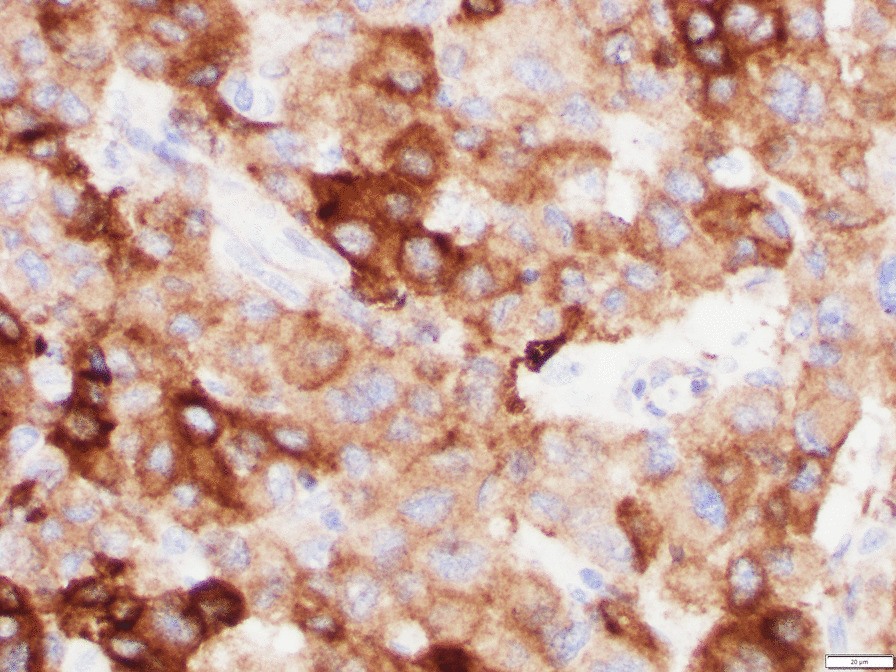
Immunostaining for synaptophysin

**Fig. 5:**
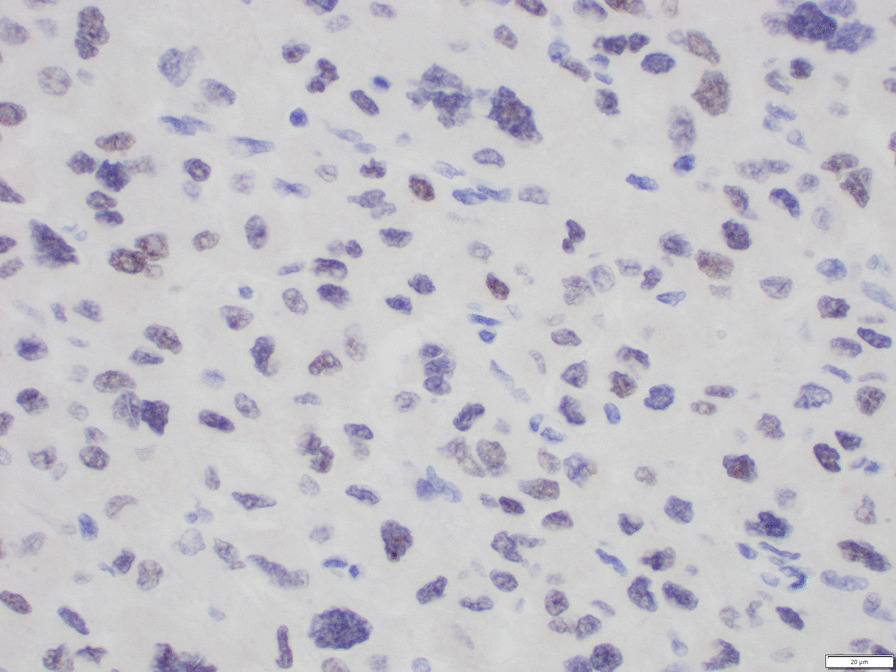
Immunostaining for steroidogenic factor 1

**Fig. 6:**
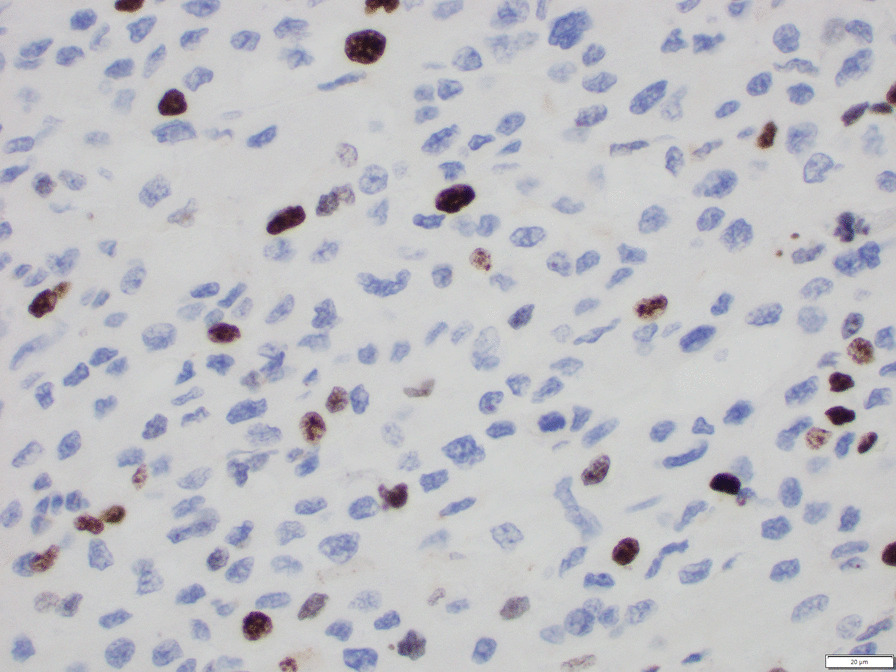
Immunostaining for Ki67 proliferation marker

Histological examination confirmed the clinical diagnosis of adrenocortical carcinoma. Neoplastic cells evidenced clear cytoplasm and atypical mitoses (Fig. 3). Immunoreactivity for synaptophysin (Fig. 4) and steroidogenic factor 1 (Fig. 5) support an adrenal cortical origin of the tumor. Ki67 proliferation index was 26.7% (Fig. 6; worse prognosis associated with index > 10%). Lymph nodes were free of malignancy.

Adjuvant chemotherapy with mitotane was begun, per standard practice to eliminate occult residual disease. Side effects included fatigue, sleepiness, malaise, and breast pain. Doses above 1.5 g per day were intolerable, and targeted plasma concentrations above 14 µg/mL have not been attained. Owing to adrenal suppression and CYP 3A4 induction by mitotane, high-dose prescription of hydrocortisone (40 mg on awakening and 20 mg 4–6 hours thereafter) proved necessary. Atorvastatin was switched to rosuvastatin.

Peripheral edema and paroxysmal dyspnea did not fully resolve until 6 months after surgery. Serum testosterone concentration increased to normal. Computed tomography of the chest and abdomen was performed on a quarterly basis to monitor for tumor.

The patient developed painful muscle cramps and restless legs. Iron deficiency was detected (3% transferrin saturation, normal 15–50%; serum ferritin concentration 9 ng/mL, normal 20–230 ng/mL). These symptoms resolved following oral iron supplementation.

Fifteen months following surgery, there has been no recurrence of tumor.

## Discussion

The etiology of the patient’s leg edema was not readily apparent. After evaluation of cardiac, hepatic, renal, and pulmonary function proved nondiagnostic, the patient’s cardiologist ordered computed tomography to rule out obstruction of venous return from the lower extremities. A massive adrenal tumor was detected, and it was indeed compressing the inferior vena cava with upstream distension. This is a very unusual presentation, prompting this case report. Although thrombus was not seen on tomographic imaging of the abdomen, pelvis, or chest, it is likely that venous thrombosis was a complication of impaired venous return and the presence of a carcinoma, causing recurrent pulmonary emboli that precipitated the paroxysmal episodes of dyspnea. The gradual remission of edema and paroxysmal dyspnea is consistent with slow resorption of clot.

Stage 3 adrenocortical carcinoma is associated with 5-year survival of 24% [4]. However, the very large size of this patient’s tumor (27 cm), the high Ki67 proliferation index (26.7%), and the presence of a satellite tumor suggest a prognosis even worse than that of “average” stage 3 disease. It was noted in retrospect that a 1.2-cm calcified right adrenal nodule was present on a computed tomographic study performed 9 years earlier, when the patient presented acutely with a calculus obstructing the right ureter. The finding of the adrenal lesion was noted in the radiology report, but was not communicated to the emergency room physician. The adrenal nodule could have been resected early for cure, or followed for progression. The literature includes one report regarding factors contributing to delayed diagnosis of adrenocortical carcinoma in patients with preexisting adrenal masses [5]. The authors reported that presumed benign status of the preexisting mass accounted for 65% of the delays in diagnosis. Failure to identify a small adrenal mass occurred in two instances, but failure to communicate the presence of a detected adrenal mass on abdominal imaging, as occurred in the current case, was not described.

Adjuvant treatment of adrenocortical carcinoma with mitotane is of unproven benefit, as randomized trials in this very rare disease are lacking. A retrospective study of 177 patients concluded that therapy has an advantage for recurrence-free survival [6], but it is possible that differences between centers other than mitotane administration confounded the findings. A systematic review concluded that mitotane therapy likely decreases recurrence and mortality after cancer resection, but that prospective controlled trials are needed [7]. In the current case, the patient suffered dose-limiting mitotane toxicity (fatigue, sleepiness, malaise, breast pain). Noxious effects of mitotane have long been recognized, with authors noting that “All of our patients found it unpleasant to take” [8]. Multiple other side effects, some serious, have been reported, including adrenal insufficiency, depression, nausea, vomiting, balance difficulties, liver damage, renal damage, and retinal damage. Palatability and bioavailability may be improved by taking this oral drug with fatty foods such as milk, chocolate, or vegetable oils. Also, the drug is known to stimulate activity of hepatic CYP 3A4 enzymes, making it necessary to alter the prescription of many concomitant medications. The current patient required high doses of hydrocortisone because of mitotane, and his serum low-density lipoprotein levels are higher after a switch from atorvastatin (a CYP 3A4 substrate) to rosuvastatin. Mitotane may also increase cholesterol levels.

The patient developed severe muscle cramps and restless legs, and was found to be profoundly iron deficient. He had mild anemia, but it was normocytic and normochromic. His surgery was extensive, lasted over 8 hours, and prompted transfusion of two units of packed red blood cells. It is likely that iron deficiency was due to surgical blood loss, a prior history of multiple blood donations, and iron lost owing to removal of the large tumor, a kidney, and one lobe of the liver.

## Conclusions

(1) Clinicians should order imaging studies of the abdomen and pelvis to rule out obstruction of venous return when evaluation of cardiac, hepatic, and renal function fails to explain the presence of peripheral edema. (2) Systems should be instituted to ensure that all abnormalities detected on imaging studies are fully communicated to the ordering physicians and their patients. This individual had a small adrenal nodule incidentally noted by the radiologist on computed tomography 9 years previously that could have been resected then, potentially saving his life, with adequate communication. (3) Randomized, controlled, double-blinded clinical studies of adjuvant mitotane therapy following resection of adrenocortical carcinoma are sorely needed to establish or refute benefit. Mitotane may be toxic, and it complicates prescription of other medications owing to induction of CYP 3A4 metabolism. (4) Iron deficiency may follow the resection of large tumors plus other organs.

## Data Availability

Not applicable.
